# 2810. A synergy test to assess the activity of the Ceftazidime-Avibactam and Aztreonam combination: An experience from a single centre in Malaysia

**DOI:** 10.1093/ofid/ofad500.2421

**Published:** 2023-11-27

**Authors:** Tang Soo Nee, Cindy Teh Shuan, Wan Nurliyana, Rina Karunakan, Helmi Sulaiman

**Affiliations:** University of Malaya, Kuala Lumpur, Kuala Lumpur, Malaysia; University of Malaya, Kuala Lumpur, Kuala Lumpur, Malaysia; International Islamic University Malaysia, Kuala Lumpur, Kuala Lumpur, Malaysia; University of Malaya, Kuala Lumpur, Kuala Lumpur, Malaysia; University of Malaya, Kuala Lumpur, Kuala Lumpur, Malaysia

## Abstract

**Background:**

Southeast Asia has a high prevalence of metallo-β-lactamase-producing carbapenem-resistant Enterobacterales (CRE). Ceftazidime-avibactam (CZA) and Aztreonam (ATM) combination is one of the preferred therapeutic options against them.

**Figure:**
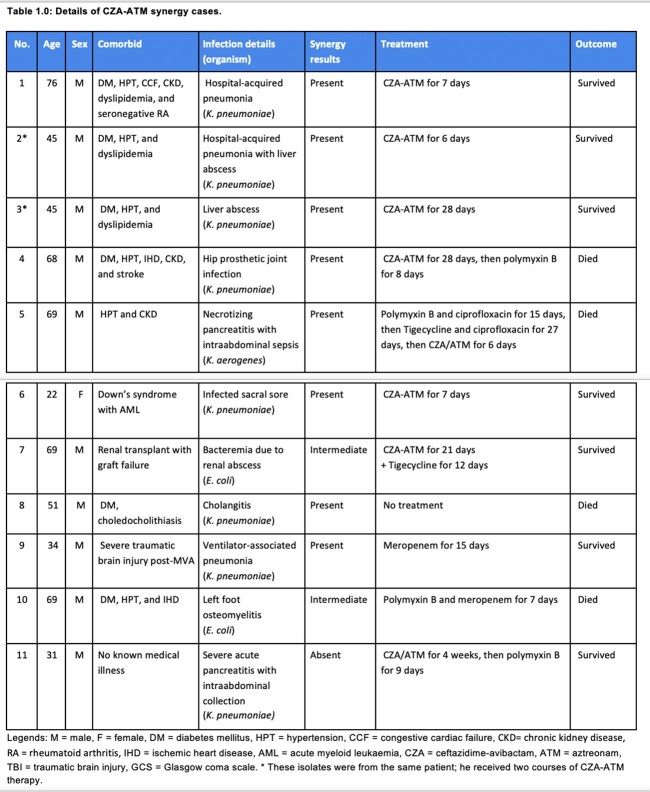

**Methods:**

A prospective study was performed at a university hospital in Kuala Lumpur, Malaysia, involving CRE isolates resistant to CZA and ATM. Susceptibility testing results were interpreted using the Clinical and Laboratory Standards Institute (CLSI) breakpoints. A synergy test for both agents [as in Falcone et al. study (CID 2021:72)] was carried out using the double disk synergy (DDS) and the gradient test superposition (GTS) methods. Synergy was considered present when the inhibition zone between the two disks became evident and if CZA reduced the ATM minimum inhibitory concentration (MIC) below its CLSI susceptibility cut-off (≤4 mg/L).

**Results:**

Eleven isolates (8 *K. pneumoniae*, 2 *E. coli*, and 1 *K. aerogenes*) from 10 patients were tested. Concordant results were seen for both methods for all the isolates. Synergy was demonstrated with 8 isolates; for 2 of the 3 isolates with no synergy, the MIC of ATM reduced to an intermediate result after GTS. The most common sites of infection were bloodstream infection (4) and intra-abdominal infection (3). Five patients received the CZA-ATM combination (one received two courses of CZA-ATM). Five isolates from four patients demonstrated synergy, and one showed intermediate synergy. The latter received additional IV tigecycline. The median time to CZA-ATM initiation from the index culture was 8 (3-13) days. The median duration of therapy was 21 (6-28) days. Four patients responded favorably to the agents (survived the infection), while the remaining patient died. For those that did not receive CZA-ATM, one received polymyxin-based therapy, one received high-dose, extended-infusion meropenem, and one received no treatment. Only the patient that received meropenem survived the infection. Please refer to Table 1.0 for more detail.

**Conclusion:**

Based on this small sample size, only a 73% synergy rate between CZA and ATM was seen for our CRE isolates resistant to CZA and ATM. Favorable treatment response was seen in those receiving the CZA-ATM combination therapy when synergy was demonstrated

**Disclosures:**

**All Authors**: No reported disclosures

